# Adaptive Periodic Speed Fluctuation Suppression for Permanent Magnet Compressor Drives

**DOI:** 10.3390/s25072074

**Published:** 2025-03-26

**Authors:** Chenchen Zhang, Yang Yang, Yimin Gong, Yibo Guo, Hongda Song, Jiannan Zhang

**Affiliations:** College of Physics, Jilin University, Changchun 130012, China; chenchenz19@mails.jlu.edu.cn (C.Z.); gongym@jlu.edu.cn (Y.G.); guoyb22@mails.jlu.edu.cn (Y.G.); songhd19@mails.jlu.edu.cn (H.S.); zhjn@jlu.edu.cn (J.Z.)

**Keywords:** speed fluctuation suppression, PMSMs, system phase shift, single-rotor compressor

## Abstract

Single-rotor compressor load torque exhibits huge periodic fluctuations, which lead to noticeable speed fluctuations. Therefore, an adaptive periodic speed fluctuation suppression method (APSFSM) based on the recursive Gauss–Newton (RGN) algorithm is proposed in this paper. The APSFSM consists of two parts: a traditional proportional-integral (PI) speed regulator is used to handle low-frequency disturbances, while the RGN part is responsible for suppressing disturbances at specific frequencies. The RGN adopts a strategy based on angle rather than time, considering the frequent speed regulation requirements of compressors, which means that it can achieve smoother speed regulation. Moreover, the RGN also has strong robustness regarding system phase shift. The compensation current of APSFSM can adaptively adjust its amplitude and phase based on real-time speed errors, maintaining the significant suppression of speed fluctuations under different operating conditions. For this paper, a series of experiments were conducted on a 650W compressor platform, and the experimental results prove the effectiveness of the proposed method.

## 1. Introduction

Permanent magnet synchronous motors (PMSMs) are widely used in the compressors of household refrigerators and air conditioners because of their high energy density and efficiency. Among these, the single-rotor compressor has been commonly adopted for low-power room air-conditioning systems because of its cost-effectiveness. However, the load torque of single-rotor compressors undergoes significant periodic fluctuations due to the gas compression and discharge processes. Specifically, during the compression phase, the load torque increases as the piston compresses the refrigerant. Conversely, during the discharge process, the load torque decreases as the piston discharges. These torque fluctuations result in notable speed variations, which, in turn, induce significant mechanical vibrations. Such vibrations not only accelerate the wear and tear of mechanical components but also generate audible noise [1,2]. Therefore, effectively compensating for fluctuating torque disturbances is a critical issue in enhancing the performance of PMSM-driven systems [3,4].

Control schemes based on the internal model principle [5] have been proposed to suppress periodic fluctuations, such as proportional resonant control (PRC) [6], repetitive control (RC) [7], and iterative learning control (ILC) [8,9]. PRC exhibits an extremely high gain at specific target frequencies, achieving near-zero steady-state errors in disturbance rejection. However, obtaining the resonant frequency inevitably involves some errors in practice. To improve robustness, PRC is often adjusted to quasi-proportional resonance controllers (QPRC) [10,11], but QPRC can only handle a narrow range of frequency variations. When there are significant changes in the resonant frequency, a direct switching strategy is employed [12], which results in a lack of compensation during the dynamic process. Adaptive resonant control (ARC), introduced in [13], uses adaptive algorithms to dynamically adjust the resonant frequency, ensuring compensation during the dynamic process. Nonetheless, in practical applications, it may still face issues with estimated frequency fluctuations. To address this problem, the authors of [14] combine a linear extended state observer with ARC, further enhancing its robustness. Additionally, phase delay is another common issue that can significantly impact the performance of QPRC. The phase adjustment method used in [15] is a fundamental adjustment that is utilized in many studies. Some studies [16,17,18] combine active disturbance rejection control (ADRC) with QPRC to further enhance and expand its application. Despite these advancements, these improved methods also increase the complexity of the setup and the computational burden.

RC and ILC utilize iterative computation methods, which reduce computational burden and design complexity. These methods leverage abundant memory units to construct an appropriate internal model and can achieve zero steady-state errors. However, when addressing the problems studied in this paper, both RC and ILC still face challenges related to frequency variation and phase delay. To address the issue of frequency variation, a varying sampling frequency is adopted in [19]. However, this approach requires hardware support and may impact other parts of the control system. In contrast, methods such as those proposed in [20,21] introduce the concept of virtual variable sampling. While this approach increases memory usage, it is more suitable for applications with narrow frequency variations. Considering the load and angle correlation of single-rotor compressors, angle-based repetitive control (ABRC) compensation methods provide a better solution for achieving speed ripple minimization at any speed and even during speed transience [22,23]. These methods generally perform well if the controlled plant has a zero-phase characteristic. Inaccurate phase compensation can weaken the suppression effect and may even affect system stability [24]. Particularly at a low sampling rate, an integer-order phase lead step may cause under-compensation or overcompensation. To achieve fine phase compensation, fractional order phase compensation methods have been proposed [25,26]. When there is an uncertain phase delay in the system, robust filtering is a commonly used method to improve convergence robustness, but it can weaken compensation performance and increase steady-state errors [27,28].

In addition, there are observer-based methods, such as the extended state observer [29], the disturbance observer [30], Kalman filters [31], etc., but these model-based methods depend on the motor parameters. There are also some engineering methods involving using the loop-up tables for compensation. This method requires extensive preliminary calibration and usually has poor robustness and versatility.

The recursive Gauss–Newton (RGN) algorithm is widely utilized in the field of signal processing for optimization purposes. In [32], a simplified RGN algorithm is used to estimate the frequency, amplitude, and phase of noisy sinusoids, addressing the serious limitations in accuracy and convergence speed faced by conventional techniques, such as adaptive linear elements and discrete or fast Fourier transforms, under conditions of sudden supply frequency drift, fundamental amplitude, or phase variations. Furthermore, RGN has been employed to address the issues of phase delay and amplitude attenuation, which are caused by phase-locked loops in traditional sensorless control, thereby improving position estimation accuracy [33]. The RGN algorithm demonstrates significant potential for addressing periodic problems.

The major contribution of this paper can be summarized as follows. Firstly, this paper proposes an APSFSM based on the RGN algorithm to address the speed fluctuation issues in single-rotor compressors. The angle-based output method ensures that the system performs well, even under conditions of frequency variation. The iterative update of amplitude and phase enables adaptive compensation for uncertain phase delay without compromising compensation accuracy. With only one parameter requiring adjustment, combined with low computational complexity and memory consumption, this method offers a better solution. Secondly, the paper includes a detailed convergence analysis of the APSFSM and provides a parameter tuning method. Additionally, it analyzes the impact of the gain and phase of the controlled plant, ensuring that the method is robust and effective under various conditions. Thirdly, experiments on a single-rotor compressor system are presented herein to demonstrate the effectiveness of the proposed method. Comparative experiments with the QPRC and ABRC methods show the superiority of the APSFSM in handling frequency variations and uncertain phase issues.

This paper is organized as follows. Section 2 introduces the fundamental mathematical models and the load characteristics of the compressor. In Section 3, taking the first harmonic as an example, the principle of the proposed method is introduced and convergence analyses are given. Moreover, the implementation considerations and parameter tuning are also elaborated upon. In Section 4, a series of experiments is conducted to verify the performance of the algorithm. Finally, our conclusions are drawn in Section 5.

## 2. Load Torque Fluctuation in Compressors

The mathematical model of PMSM in the synchronous rotating coordinate system is established as follows:

(1) $$\left\{\begin{array}{l} u_{d}=R_{s}i_{d}+L_{d}\frac{di_{d}}{dt}-\omega_{e}L_{q}i_{q}\\ u_{q}=R_{s}i_{q}+L_{q}\frac{di_{q}}{dt}+\omega_{e}L_{d}i_{d}+\omega_{e}\lambda_{f} \end{array}\right.$$

(2) $$\left\{\begin{array}{l} J\frac{d\omega_{m}}{dt}=T_{e}-T_{L}-B\omega_{m}\\ T_{e}=\frac{3}{2}P\left(\lambda_{f}+\left(L_{d}-L_{q}\right)i_{d}\right)i_{q} \end{array}\right.$$

where $u_{d}$ and $u_{q}$ are the stator voltages in the *dq* axes,$\;i_{d}$ and $i_{q}$ are the stator current in the *dq* axes, $R_{s}$ is the stator resistance, $L_{d}$ is the *d*-axis inductance, $L_{q}$ is the *q*-axis inductance, $\omega_{e}$ is the rotor’s electrical speed, and $\lambda_{f}$ is the permanent magnet flux linkage. J is the inertia, B is the friction coefficient, $\omega_{m}$ is the mechanical speed, $T_{e}$ is the motor electromagnetic torque, $T_{L}$ is the motor load torque, and P is the number of pole pairs.

In the $i_{d}=0$ control strategy, this is:

(3) $$T_{e}=\frac{3}{2}P\lambda_{f}i_{q}=K_{t}i_{q}$$

where $K_{t}$ is the torque constant.

**Figure 1:**
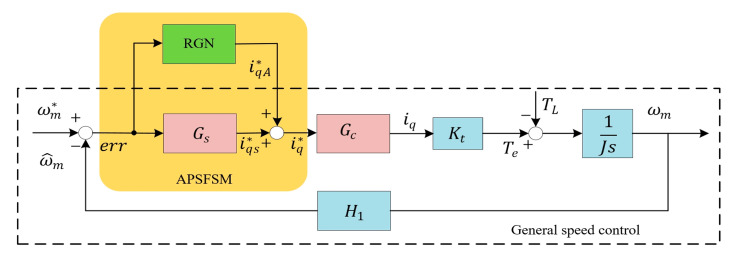
The speed control block diagram.

Based on the mathematical models, the general speed control block diagram can be depicted as shown in the dotted box in Figure 1. Here, $\omega_{m}^{*}$ is the speed command, $\omega_{m}$ is the actual motor speed, ${\hat{\omega }}_{m}$ is the obtained speed, err is the speed error, $G_{s}$ and $G_{c}$ represent the transfer functions of the speed regulator and current regulator, respectively. $i_{q}^{*}$ is the *q*-axis current command, $i_{qA}^{*}$ is the compensation current command of RGN, $i_{qs}^{*}$ is the current command of the speed regulator, and $H_{1}$ represents the transfer function of speed acquisition.

**Figure 2:**
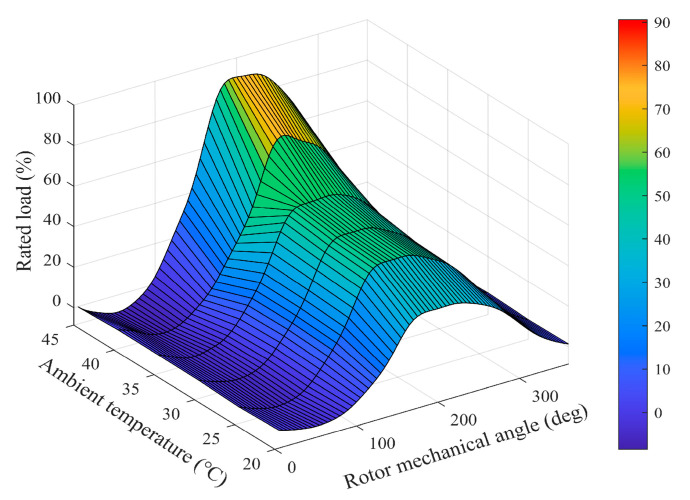
Characteristics of load torque of the single-rotor compressor.

In practical applications, the speed regulator and current regulator can generally be equated to first-order low-pass filters. Typically, the bandwidth of the speed regulator $\omega_{c\_c}$ is set much larger, while the value of the bandwidth of the current regulator $\omega_{c\_s}$ is set smaller. With this control design, general speed regulation performance can be satisfied. However, the single-rotor compressor exhibits significant periodic fluctuations in $T_{L}$ during operation, due to its unique mechanical structure, with a frequency equal to $\omega_{m}$. Figure 2 shows the waveform of $T_{L}$ varying with the rotor mechanical angle $\theta_{m}$. It is worth noting that $T_{L}$ is also affected by ambient temperature. However, changes in ambient temperature are usually slow.

The periodically varying $T_{L}$ can be expressed in Fourier series expansion as in Equation (4):

(4) $$T_{L}(k)=T_{L0}\left(k\right)+T_{L1}\left(k\right)+O_{TL}\left(k\right)$$

where k=1,2,…,N. $T_{L0}\left(k\right)$ is the DC component, $T_{L1}\left(k\right)$ is the first-order harmonic, and $O_{TL}(k)$ represents the higher-frequency components.

Although the general speed regulator is sufficient to deal with some low-frequency disturbances in the loop, it becomes problematic when facing the challenge of high-frequency $T_{L}$, thus leading to noticeable speed fluctuations and noise issues. The compressor speed $\omega_{m}$ varies periodically due to the fluctuating $T_{L},$ which can be expressed as:

(5) $$\omega_{m}\left(k\right)=\omega_{m0}\left(k\right)+\omega_{m1}\left(k\right)+O_{\omega m}\left(k\right)$$

where $\omega_{m0}\left(k\right)$ is the DC component, $\omega_{m1}\left(k\right)$ is the first-order harmonic, and $O_{\omega m}O(k)$ is the higher-frequency component. Among these, $\omega_{m1}\left(k\right),$ caused by $T_{L1}\left(k\right),$ dominates [34], and compensating only the first harmonic is an efficient and universal approach in practical applications.

## 3. Proposed Method

### 3.1. The APSFSM

To address the above issue, an additional path using the RGN method is employed to process $T_{L1}$, while leaving the low-frequency disturbances to the conventional $G_{s}$. The basic control diagram is shown as the shaded part of Figure 1.

With generality, the gain and phase shift of the control plant at the frequency of $\omega_{m}$ can be defined as:

(6) $$K=\left\|{K_{t}G}_{c}H_{1}/J_{s}\left(\omega_{m}\right)\right\|=K_{t}K_{c}K_{H}K_{J}$$

(7) $$\rho =∠{K_{t}G}_{c}H_{1}/J_{s}\left(\omega_{m}\right)=\rho_{c}+\rho_{H}+\rho_{J}$$

where $K_{c}$, $K_{H}$, and $K_{J}$ are the gains and $\rho_{c}$, $\rho_{H}$, and $\rho_{J}$ are the phase shifts of $G_{c}$, $H_{1}$, and $1/J_{s}$ respectively.

Since the waveform of $T_{L}$ is strongly correlated with $\theta_{m}$, the form of $T_{L1}$ in math can be written as follows:

(8) $$\begin{array}{l} T_{L1}\left(k\right)=A\sin\left(\theta_{m}\left(k\right)+\varphi \right)\\ \;\;\;\;\;\;\;\;\;\;\;\;=BK_{c}\sin\left(\theta_{m}\left(k\right)+\varphi_{c}\right)+CK_{c}\cos\left(\theta_{m}\left(k\right)+\varphi_{c}\right)\# \end{array}$$

where $\theta_{m}(k)$ is the $\theta_{m}$ at execution time *k*; A and φ are the amplitude and phase of $T_{L1}$. Due to the slow temperature change, the effect of A and φ when varied are ignored in the subsequent analysis. For convenience, $T_{L1}$ can be further written as the sum of a sine signal with amplitude B and a cosine signal with amplitude C, as shown in Equation (8).

Then, the compensation component of $T_{L1}$ can be computed as $i_{qA1}^{*}$, according to Equation (3):

(9) $$i_{qA1}^{*}(k)=\hat{B}\left(k-1\right)sin\theta_{m}\left(k\right)+\hat{C}\left(k-1\right)cos\theta_{m}\left(k\right)$$

where $\hat{B}$ and $\hat{C}$ represent the amplitudes of the sine and cosine components of $i_{qA1}^{*}$, respectively. It is worth noting that, for simplicity in the following expressions, the phase effect of $G_{c}$ has already been accounted for in Equation (8), and there is only a coefficient $K_{c}K_{t}$ between B, C and $\hat{B}$, $\hat{C}$.

Then, the speed error is:

(10) $$err(k)=\omega_{m}^{*}-\left(G_{c}K_{t}i_{q}^{*}(k)-T_{L}(k)\right)\frac{H_{1}}{Js}$$

where $i_{q}^{*}\left(k\right)=i_{qA1}^{*}\left(k\right)+i_{qs}^{*}\left(k\right)$, as shown in Figure 1. The cost function is selected as follows:

(11) $$\varepsilon \left(k\right)=\sum_{i=0}^{k}\lambda^{k-i}{err}^{2}\left(k\right)$$

where 0<λ<1 is the forgetting factor.

When ε(k) has a minimum value, the optimal compensation of the first-order harmonic is achieved. Let the parameter vector be $\hat{v}\left(k\right)={\left[\hat{B}\left(k\right)\;\hat{C}\left(k\right)\right]}^{T}$.

According to the RGN method [31], the updating equations are given by Equation (12):

(12) $$\hat{v}\left(k\right)=\hat{v}\left(k-1\right)-H^{-1}\left(k\right)\Phi \left(k\right)err\left(k\right)$$

(13) $$H\left(k\right)=\sum_{i=0}^{k}\lambda^{k-i}\Phi \left(i\right){\Phi \left(i\right)}^{T}$$

where H is the Hessian matrix, and Φ is the gradient vector.

Substituting Equation (9) into Equation (10), err(k) can be rearranged as:

(14) $$\begin{array}{l} err\left(k\right)=-K\hat{B}\left(k-1\right)sin{(\theta }_{m}\left(k\right)+\rho )\\ -K\hat{C}\left(k-1\right)cos(\theta_{m}\left(k\right)+\rho )+O\# \end{array}$$

where $O=\omega_{m}^{*}+T_{L}\left(k\right)H_{1}/Js$ represents the components unrelated to $\hat{v}\left(k\right)$.

In this case, the gradient vector is given by Equation (15), and the Hessian matrix is shown in Equation (16).

(15) $$\Phi \left(k\right)=\frac{\partial err\left(k\right)}{\partial v}=K\left[\begin{array}{c} -sin{(\theta }_{m}\left(k\right)+\rho )\\ -\cos\left(\theta_{m}\left(k\right)+\rho \right) \end{array}\right]$$

$H\left(k\right)$ can be approximated by (16) when $\theta_{m}\left(k\right)-\theta_{m}\left(k-1\right)$ is not close to 0 or π. The inverse Hessian matrix can, therefore, be determined as:

(16) $$\;\begin{array}{l} H\left(k\right)=\sum_{i=0}^{k}\lambda^{k-i}K^{2}\left[\begin{array}{cc} {sin}^{2}{(\theta }_{m}\left(k\right)+\rho ) & \frac{\sin\left({2\theta }_{m}\left(k\right)+2\rho \right)}{2}\\ \frac{\sin\left({2\theta }_{m}\left(k\right)+2\rho \right)}{2} & {cos}^{2}{(\theta }_{m}\left(k\right)+\rho ) \end{array}\right]\\ \approx \sum_{i=0}^{k}\lambda^{k-i}K^{2}\left[\begin{array}{cc} \frac{1}{2} & 0\\ 0 & \frac{1}{2} \end{array}\right]\\ =\frac{1-\lambda^{k+1}}{2\left(1-\lambda \right)}K^{2}\left[\begin{array}{cc} 1 & 0\\ 0 & 1 \end{array}\right]\# \end{array}$$

(17) $${H\left(k\right)}^{-1}=1/c\left(k\right)\left[\begin{array}{cc} 1 & 0\\ 0 & 1 \end{array}\right]$$

(18) $$c\left(k\right)=\frac{1-\lambda^{k+1}}{2\left(1-\lambda \right)}K^{2}$$

The updating equation of $c\left(k\right)$ can be written as:

(19) $$c\left(k\right)=(\lambda c\left(k-1\right)+\frac{1}{2})K^{2}$$

Substituting Equations (15), (17) and (18) into Equation (12) yields the following updating Equation (20):

(20) $$\left[\begin{array}{c} \hat{B}\left(k\right)\\ \hat{C}\left(k\right) \end{array}\right]=\left[\begin{array}{c} \hat{B}\left(k-1\right)+K\sin\left(\theta_{m}\left(k\right)+\rho \right)*err(k)/c(k)\\ \hat{C}\left(k-1\right)+K\cos\left(\theta_{m}\left(k\right)+\rho \right)*err(k)/c(k) \end{array}\right]$$

Substituting the iterative result of Equation (20) into Equation (9) reveals the compensation current of $T_{L1}$.

### 3.2. The Convergence Analysis

#### 3.2.1. Convergence Proof

The APSFSM consists of two parts: $G_{s}$, which is responsible for suppressing low-frequency disturbances, and the RGN, which is responsible for high-frequency disturbances. By setting $\omega_{c\_s}\leq \omega_{m}/10$, interference between the two parts can be neglected. Therefore, the convergence analysis can also be performed separately. The rest of the analysis focuses on the convergence properties of the RGN part.

Let the initial values be zero:

(21) $$\hat{B}\left(0\right)=\hat{C}\left(0\right)=0$$

Equation (20) can be written in the following accumulated form:

(22) $$\left[\begin{array}{c} \hat{B}\left(k\right)\\ \hat{C}\left(k\right) \end{array}\right]=K\sum_{i=0}^{k}\left[\begin{array}{c} \sin\left(\theta_{m}\left(i\right)+\rho \right)*err\left(i\right)/c\left(i\right)\\ \cos\left(\theta_{m}\left(i\right)+\rho \right)*err\left(i\right)/c\left(i\right) \end{array}\right]$$

Equation (22) is similar to a discrete Fourier transform. When k is large enough, it is reasonable to assume that $\hat{B}\left(k\right)$ and $\hat{C}\left(k\right)$ are only affected by the first-order harmonic ${err}_{1}(k)$ in err(k). Moreover, the RGN output (9) also only affects the first-order harmonic in the system. Therefore, err(i) in Equation (22) can be replaced by ${err}_{1}\left(i\right)$.

Based on Equation (10):

(23) $${err}_{1}\left(k\right)=-\frac{H_{1}}{Js}\left(T_{L1}\left(k\right)-G_{c}K_{t}i_{qA1}^{*}\left(k\right)-G_{c}K_{t}i_{qs1}^{*}\left(k\right)\right)$$

$i_{qs1}^{*}$ represents the $\omega_{m}$-synchronous component of the $G_{s}$ output.

If ${err}_{1}$ has the following form:

(24) $${err}_{1}\left(k+1\right)=\gamma {err}_{1}\left(k\right)$$

and $\left\|\gamma \right\|<1$, then ${err}_{1}$ will converge during the iteration process.

Since $\omega_{c\_s}\leq \omega_{m}/10$, the bandwidth limitation of the current regulator ensures that the value of $i_{qs1}^{*}$ is negligible, resulting in $i_{qs1}^{*}\left(k\right)\approx 0$. Substituting Equations (6)–(9) into Equation (23) yields:

(25) $$\begin{array}{l} {err}_{1}\left(k\right)=K\left(B-\hat{B}\left(k-1\right)\right)\sin\left(\theta_{m}\left(k\right)+\rho \right)\\ +K\left(C-\hat{C}\left(k-1\right)\right)\sin\left(\theta_{m}\left(k\right)+\rho \right)\# \end{array}$$

It should be noted that:

(26) $$\begin{array}{l} sin\theta_{m}\left(k+1\right)=sin\theta_{m}\left(k\right)+\frac{dsin\theta_{m}\left(k\right)}{dt}T_{s}\\ =sin\theta_{m}\left(k\right)+\omega_{m}T_{s}cos\theta_{m}\left(k\right)\# \end{array}$$

where $T_{s}$ is the execution period.

Similarly:

(27) $$cos\theta_{m}\left(k+1\right)=cos\theta_{m}\left(k\right)-\omega_{m}T_{s}sin\theta_{m}\left(k\right)$$

Then, based on Equations (25)–(27), it follows that:

(28) $${err}_{1}\left(k+1\right)-{err}_{1}\left(k\right)=\alpha \left(k\right)+\beta \left(k\right)$$

where:

(29) $$\begin{array}{l} \alpha \left(k\right)=K\sin\left(\theta_{m}\left(k\right)+\rho \right)\left(\hat{B}\left(k-1\right)-\hat{B}\left(k\right)\right)\\ +K\cos\left(\theta_{m}\left(k\right)+\rho \right)\left(\hat{C}\left(k-1\right)-\hat{C}\left(k\right)\right) \end{array}$$

(30) $$\begin{array}{c} \beta \left(k\right)=K\omega_{m}T_{s}\cos\left(\theta_{m}\left(k\right)+\rho \right)\left(B-\hat{B}\left(k\right)\right)\\ -K\omega_{m}T_{s}\sin\left(\theta_{m}\left(k\right)+\rho \right)\left(C-\hat{C}\left(k\right)\right) \end{array}$$

Substituting Equations (18) and (20) into Equation (29) yields:

(31) $$\alpha \left(k\right)=-\frac{2\left(1-\lambda \right)}{1-\lambda^{k+1}}{err}_{1}\left(k\right)$$

Substituting (20) into (30) and rearranging them yields:

(32) $$\begin{array}{l} \beta \left(k\right)=K\omega_{m}T_{s}\cos\left(\theta_{m}\left(k\right)+\rho \right)\left(B-\hat{B}\left(k-1\right)\right)\\ -K\omega_{m}T_{s}\sin\left(\theta_{m}\left(k\right)+\rho \right)\left(C-\hat{C}\left(k-1\right)\right) \end{array}$$

We define the operator P:

(33) $$\left\|P\right\|=1$$

(34) $$∠P=\frac{\pi }{2}$$

Then:

(35) $$\beta \left(k\right)=\omega_{m}T_{s}{err}_{1}\left(k\right)P$$

When substituting Equations (31) and (35) into (28), the result can be obtained as:

(36) $$\gamma =1-\frac{2\left(1-\lambda \right)}{1-\lambda^{k+1}}+\omega_{m}T_{s}P$$

Since 0<λ<1, if k is large enough, it is easy to obtain the following result:

(37) $$\left\|\gamma \right\|=\sqrt{{\left(2\lambda -1\right)}^{2}+{\left(\omega_{m}T_{s}\right)}^{2}}$$

By substituting the convergence condition $\left\|\gamma \right\|<1$, this yields:

(38) $${\left(2\lambda -1\right)}^{2}+{\left(\omega_{m}T_{s}\right)}^{2}<1$$

Therefore, the proposed APSFSM converges with a suitable λ, according to Equation (38).

#### 3.2.2. Implementation Considerations

In the application of the proposed APSFSM, it is necessary to substitute the actual system parameters K and ρ into Equation (20). A prepared lookup table with $\omega_{m}$ as the input can be a good choice. However, accurately acquiring the parameters K and ρ is not easy. Incorrect values would affect the performance of the proposed method.

**Figure 3:**
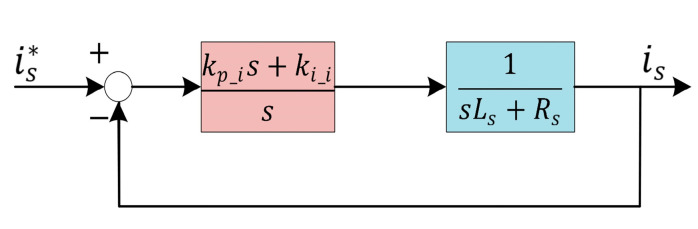
A typical current regulator model.

The current loop is chosen as an example to illustrate this problem. The current regulator model under ideal conditions is shown in Figure 3, where $i_{s}^{*}$ is the current command, $k_{p\_i}$ is the proportional gain coefficient, $k_{i\_i}$ is the integral gain coefficient, $i_{s}$ the actual current, and $L_{s}$ is the motor inductance. Regardless of the back EMF term, we let $k_{p\_i}=L_{s}*\omega_{c\_c}$ and $k_{i\_i}=R_{s}*\omega_{c\_c}$; then, the transfer function is:

(39) $$\frac{i_{s}}{i_{s}^{*}}=\frac{\omega_{c\_c}}{s+\omega_{c\_c}}$$

**Figure 4:**
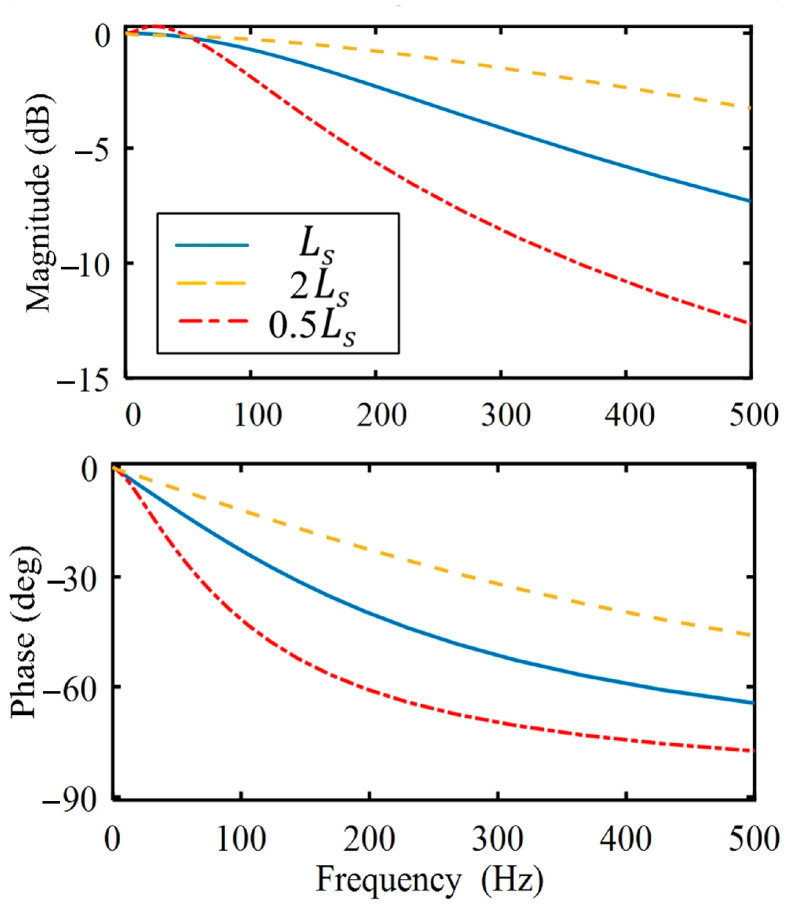
Bode diagrams of the current loop for different $L_{s}$ values.

However, the parameters of the motor would change along with the working conditions. Figure 4 shows the bode diagrams of the current loop for different $L_{s}$ values. It is not difficult to see that the magnitude and phase are influenced not only by the frequency but also by the $L_{s}$.

The other parts of the system may also face similar issues. Therefore, Equation (20) should be expressed in a more accurate form as Equation (40):

(40) $$\left[\begin{array}{c} \hat{B}\left(k\right)\\ \hat{C}\left(k\right) \end{array}\right]=\left[\begin{array}{c} \hat{B}\left(k-1\right)+\hat{K}\sin\left(\theta_{m}\left(k\right)+\hat{\rho }\right)*err(k)/c(k)\\ \hat{C}\left(k-1\right)+\hat{K}\cos\left(\theta_{m}\left(k\right)+\hat{\rho }\right)*err\left(k\right)/c\left(k\right) \end{array}\right]$$

where $\hat{K}$ and $\hat{\rho }$ are the adopted values of K and ρ.

Substituting Equation (40) into (28)–(30) and recalculating, Equation (36) is updated to Equation (41):

(41) $$\gamma =1-\frac{2\left(1-\lambda \right)}{1-\lambda^{k+1}}*\frac{\hat{K}}{K}\cos\left(\rho -\hat{\rho }\right)+\omega_{m}T_{s}P$$

Therefore, the values of $\hat{K}$ and $\hat{\rho }$ must also meet certain requirements to ensure that the convergence condition $\left\|\gamma \right\|<1$ is still satisfied. Considering the most general case where $\omega_{m}T_{s}$ is very small and λ is close to 1, generally, the influence of $\hat{K}/K$ is relatively small. It is always possible to adjust the value of λ to offset the impact of the inaccurate $\hat{K}$.

However, the influence of $\hat{\rho }$ is more significant, specifically, when $\cos\left(\rho -\hat{\rho }\right)\leq 0$, $\left\|\gamma \right\|$ would be larger than 1, causing the algorithm to diverge. Therefore, it is essential to ensure that the difference between $\hat{\rho }$ and ρ does not approach π/2.

In order to improve the ability of the actual current to follow $i_{qA}^{*}$ and to simplify the calculation by neglecting the influence of the current regulator during the acquisition of $\hat{K}$ and $\hat{\rho }$, a simple feedforward will be added to the traditional PI current regulator in this paper.

(42) $$\left\{\begin{array}{l} u_{d_{ff}}={-\omega }_{e}L_{q}i_{qA}^{*}\\ u_{q_{ff}}=R_{s}i_{qA}^{*}+pL_{q}i_{qA}^{*} \end{array}\right.$$

**Figure 5:**
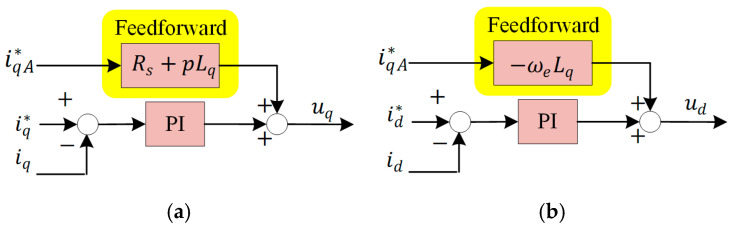
The current regulator with feedforward: (**a**) d-axis; (**b**) q-axis.

The feedforward component is shown in Equation (42), and the specific implementation is shown in Figure 5**.**

#### 3.2.3. Parameter Tuning of the Proposed Compensation Method

The convergence performance of the proposed method is determined by the attenuation rate of ${err}_{1}$. When the $\hat{K}$ and $\hat{\rho }$ deviations are small and $\omega_{m}T_{s}$ is constant, it can be seen from Equations (24) and (38) that a faster convergence rate can be achieved when λ is close to 0.5. However, the sensitivity to high-frequency noise is also increased at the same time. Conversely, when λ is close to 1, this results in a lower convergence rate but better high-frequency noise rejection properties. Considering a practical application where the compressor load fluctuates mainly periodically with the $\theta_{m}$ and does not have strong dynamic response requirements, a larger λ value is suggested.

Regarding the initial value setting of $\hat{B}\left(0\right)$ and $\hat{C}\left(0\right)$, theoretically random initial values are all supported. However, in practice, for the compressor system studied in this paper, excessively wrong initial values may exacerbate the speed fluctuation problem directly. Therefore, it is recommended to set the values to 0.

## 4. Experimental Verification

**Figure 6:**
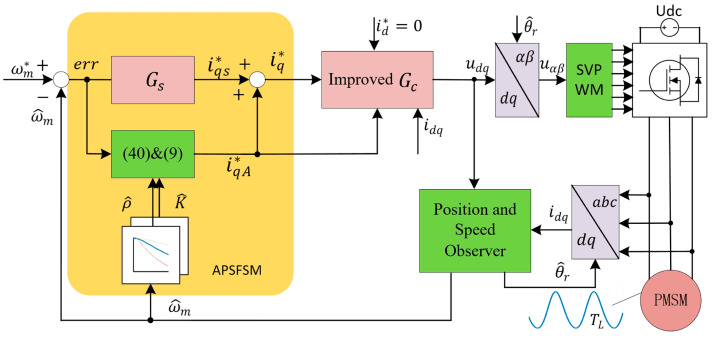
The final overall control block diagram.

**Figure 7:**
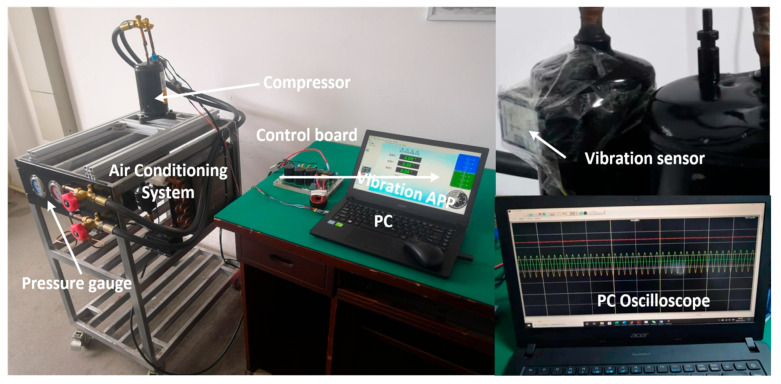
The experimental setup.

The final overall control block diagram is shown in Figure 6 and the experimental setup is shown in Figure 7. The compensation scheme is realized on a Renesas R5F24T8ADFM chip. The switching frequency of the power devices, the algorithm execution frequency, and the sampling frequency of the system are all 8 kHz. The compressed parameters are shown in Table 1, and the air conditioning system, which uses R410a refrigerant, provides a true load environment for the compressor. Two pressure gauges are used to measure the pressure on the suction side and the exhaust side. To better quantify the performance of the proposed algorithm, a vibration sensor (WT901C) monitors the compressor vibration. All experimental data are transmitted to a PC via serial communication, and the sole parameter requiring adjustment in the APSFSM, λ, is experimentally set to 0.95. Additionally, comparative experiments with QPRC and ABRC are conducted. Notably, the phase compensation of QPRC and ABRC adopts the same method as APSFSM, as depicted in Figure 6. The key parameters of QPRC are a proportional coefficient of 0.7, a resonant coefficient of 200, a bandwidth of 0.2% ${\hat{\omega }}_{m}$, and a resonant frequency of ${\hat{\omega }}_{m}$. ABRC uses 512 memory units, a forgetting factor of 0.995, and the robust filtering uses 5 adjacent units.

### 4.1. Basic Compensation Result Analysis

**Figure 8:**
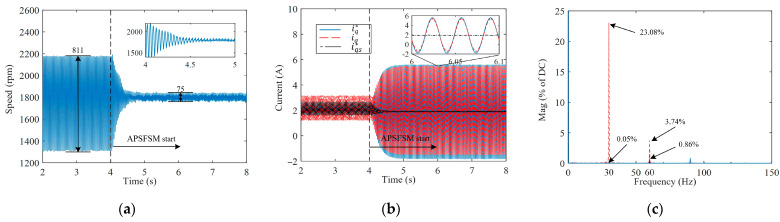
Experimental results at 1800 rpm: (**a**) the motor speed; (**b**) the q-axis current; (**c**) the FFT of the speed with/without APSFSM at 1800 rpm.

Figure 8 shows the experimental results when focusing on a speed of 1800 rpm. Figure 8a illustrates that under general speed control, the speed fluctuates up to 811 rpm. Upon the activation of APSFSM, the speed fluctuation diminishes to 75 rpm within approximately 0.6 s. Notably, Figure 8b depicts the q-axis current waveform, demonstrating a smooth convergence process, devoid of abnormal oscillations. Additionally, the locally amplified waveform of the q-axis current underscores the compensation effect, as evidenced by the near-linear output $i_{qs}^{*}$ of $G_{s}$, which is responsible for balancing the DC component in $T_{L}$, and RGN’s role in balancing $T_{L1}$. Furthermore, under the influence of the enhanced $G_{c}$ control, $i_{q}$ can follow $i_{q}^{*}$ well. Figure 8c shows the FFT results of speed. Using the proposed APSFSM, the first harmonic is reduced from 23.8% to 0.05%. While the second and third harmonics change due to the nonlinearity of the compressor system, APSFSM can effectively suppress the target harmonic content in the speed.

**Figure 9:**
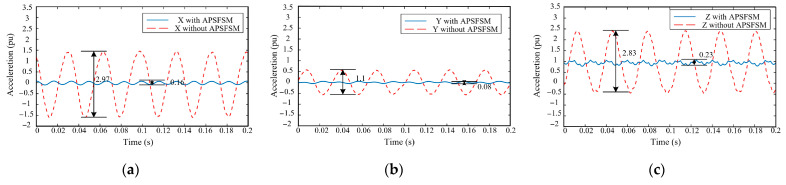
The vibration results in XYZ with/without APSFSM at 1800 rpm: (**a**) *x*-axis; (**b**) *y*-axis; (**c**) *z*-axis.

Moreover, the amelioration of speed fluctuations through APSFSM translates into an improvement in vibration issues. The three-dimensional vibration results in XYZ are shown in Figure 9a–c. The data is normalized using gravitational acceleration. With APSFSM, the fluctuation of the *x*-axis decreased from 2.97 to 0.16, the result of the *y*-axis decreased from 1.1 to 0.08, and the result of the *z*-axis decreased from 2.83 to 0.2.

**Figure 10:**
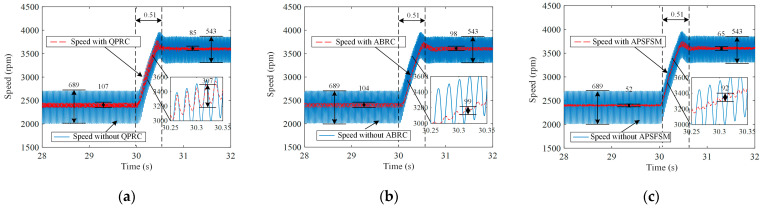
Experimental results of the different methods under the speed step-up: (**a**) QPRC; (**b**) ABRC; (**c**) APSFSM.

**Figure 11:**
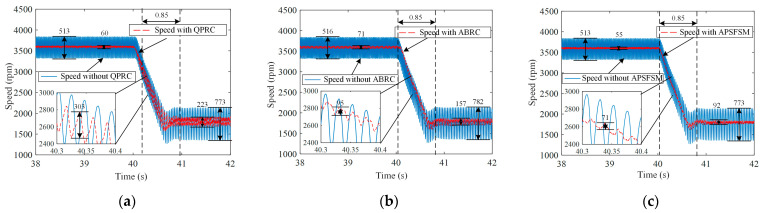
Experimental results of different methods under the speed step-down: (**a**) QPRC; (**b**) ABRC; (**c**) APSFSM.

Speed waveforms under various control methods during speed step transitions are recorded in Figure 10 and Figure 11. It takes 0.51 s for the speed to increase from 2400 rpm to 3600 rpm, and it takes 0.85 s for the speed to decrease from 3600 rpm to 1800 rpm. A comparative analysis during these speed transitions reveals that ABRC and APSFSM consistently maintain low-speed fluctuations, whereas QPRC exhibits significantly higher fluctuations. From the zoomed-in sections of Figure 10 and Figure 11, it can be observed that during the speed increase, the fluctuations for QPRC, ABRC, and APSFSM are 307 rpm, 99 rpm, and 92 rpm, respectively. Similarly, during the speed decrease, the fluctuations are 303 rpm, 95 rpm, and 71 rpm, respectively. The experimental results demonstrate that the angle-based APSFSM performs exceptionally well under frequency variations.

**Figure 12:**
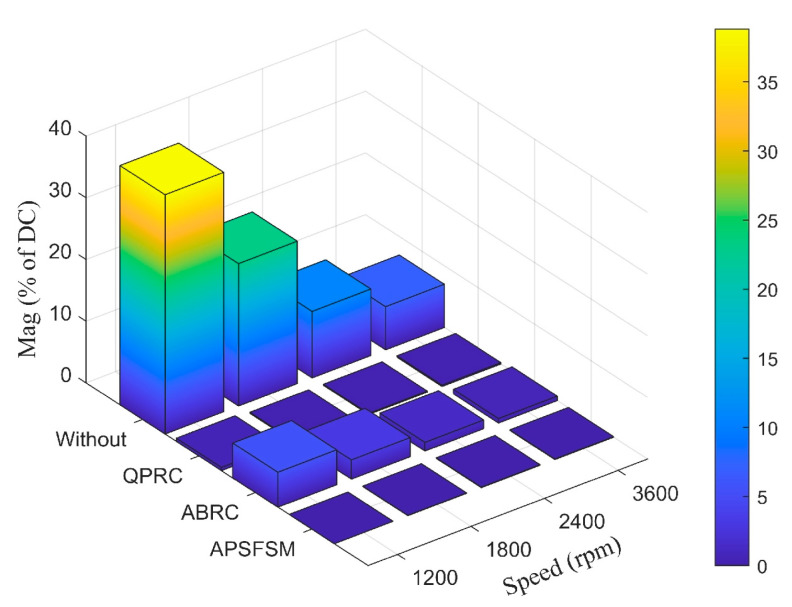
The first harmonic FFT results from the different methods at various speeds.

Figure 12 presents the first harmonic FFT results with different methods at various speeds. Without any compensation, the disturbance of the first harmonic reaches a maximum of 38.79% at 1200 rpm and decreases with the increase in speed. After applying the compensation methods, the first harmonic components are significantly reduced. Among them, QPRC and APSFSM maintain low levels across all speeds, while ABRC performs worse at low speeds. In order to describe this phenomenon more intuitively, Table 2 and Table 3 provide more detailed information. Table 2 shows the pressure values during the experiment. Since the balance pressure of the suction side and the exhaust side of the compressor compensation system varies at different speeds, the results reflect different loads. Table 3 provides detailed statistics of the first harmonics at different speeds. The range of the first harmonic under APSFSM control is between 0.01% and 0.08%, while the corresponding range of QPRC is between 0.16% and 0.52%. Although, in theory, all three methods can achieve nearly perfect harmonic suppression, the parameter adjustment of QPRC and ABRC must consider the effects of frequency changes and the system’s uncertain phase shifts. Therefore, the proposed APSFSM demonstrates better performance in practical applications.

### 4.2. Robustness Analysis

**Figure 13:**
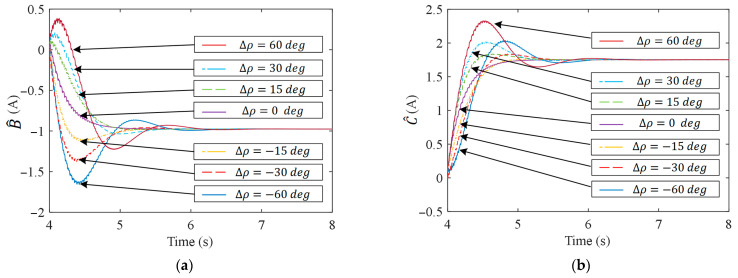
Experimental results of APSFSM with ∆ρ: (**a**) results of $\hat{B}$; (**b**) results of $\hat{C}$.

**Figure 14:**
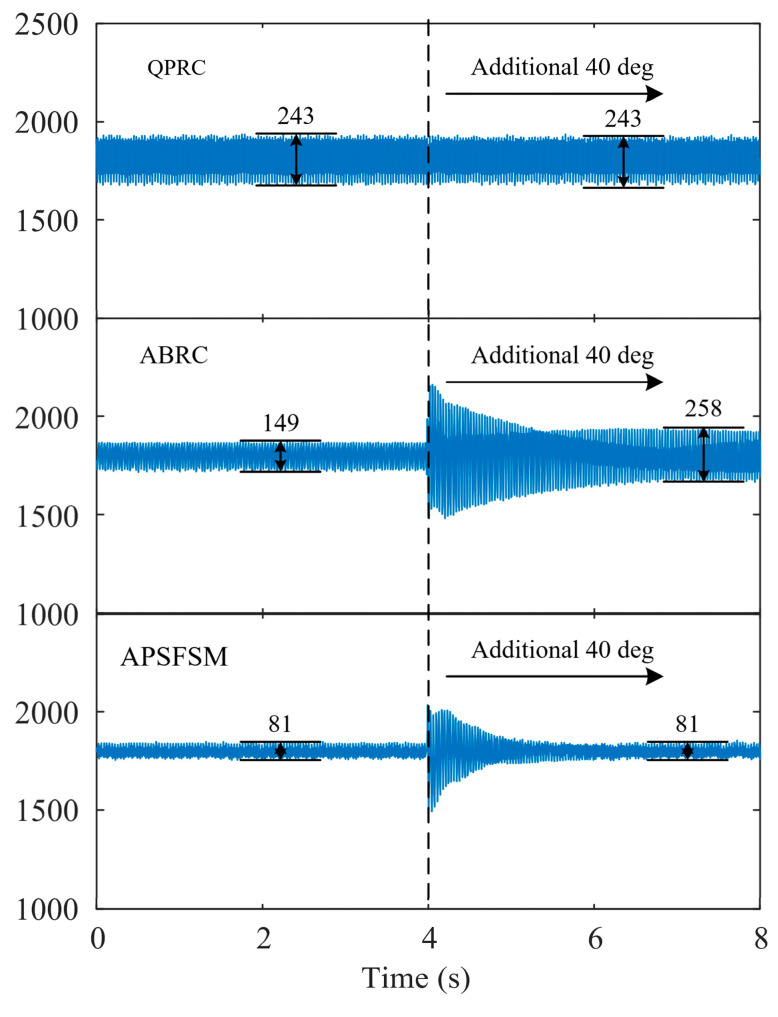
Experimental results of different methods with an additional 40 deg.

To verify the robustness of APSFSM regarding system phase delay, ∆ρ is added to $\hat{\rho }$ in Figure 6. Figure 13 shows the experimental results with varying ∆ρ values. Figure 13a,b, respectively, show the waveforms of $\hat{B}$ and $\hat{C}$ over time. It can be seen that when there is no additional angle, the convergence rate of $\hat{B}$ and $\hat{C}$ is the fastest, with no overshoot. As ∆ρ increases, the overshoot becomes larger and the convergence time is longer, but the final convergence result remains unchanged. Figure 14 shows the results of different methods when ∆ρ=40 deg. The speed fluctuation of ABRC increases from 149 rpm to 258 rpm after the additional ∆ρ is applied, causing the system to gradually diverge. However, the speed fluctuation of APSFSM and QPRC remains unchanged. The experimental results show that even with the robust filtering in ABRC, its phase margin remains limited, whereas APSFSM exhibits greater robustness to uncertain phase delays.

**Figure 15:**
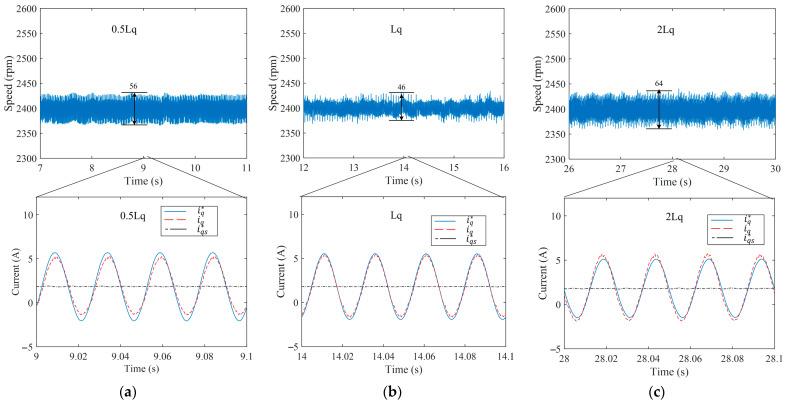
Experimental results with the inductance variation: (**a**) results with ${0.5L}_{q}$; (**b**) results with $L_{q}$; (**c**) results with ${2L}_{q}$.

Figure 15 shows the experimental results with inductance variation. When the inductance changes, the current control performance changes dramatically. With an accurate $L_{q}$, $i_{q}$ follows $i_{q}^{*}$ well. However, when $L_{q}$ is reduced to ${0.5L}_{q}$, $i_{q}$ lags behind $i_{q}^{*}$, with significantly attenuated amplitude. Fortunately, the proposed method can automatically adjust the amplitude and phase of $i_{q}^{*}$ to achieve the same final control effect. As shown in the current results, the final results of $i_{q}$ are roughly the same. Additionally, after compensation, the speed fluctuations for the three different inductance parameters are 56 rpm, 46 rpm, and 64 rpm, respectively, indicating that the proposed APSFSM maintains performance consistency and robustness to parameter changes.

## 5. Conclusions

In this paper, an adaptive periodic speed fluctuation suppression method (APSFSM) is proposed for single-rotor compressors. The core concept of the method is to eliminate low-frequency disturbances using a conventional speed regulator while employing the RGN method in a parallel path to address disturbances at specific frequencies. Angle-based compensation, as opposed to time-based, provides significant advantages under variable speed conditions. Furthermore, both the theoretical analysis and experimental results demonstrate that this method exhibits strong robustness to system phase shifts. The experimental results on the compressor drive system confirm the effectiveness of the proposed APSFSM, and its comprehensive performance is better than that of quasi-proportional resonance controllers (QPRC) and angle-based repetitive control (ABRC).

## Figures and Tables

**Table 1:** Compressor parameters.

Symbol	Parameter	Value with Unit
$P_{rated}$	Rated power	650 W
$V_{rated}$	Rated voltage	AC 220 V
P	Number of pole pairs	3
$\omega_{m\_rated}$	Rated rotor speed	3600 rpm
$\omega_{m\_min}$	Minimum speed	900 rpm
$R_{s}$	Stator resistance	0.825 Ω
$L_{d}$	*d*-axis inductance	11.4 mH
$L_{q}$	*q*-axis inductance	15.2 mH
J	Inertia constant	0.000286 kg $m^{2}$
$K_{t}$	Torque constant	0.45 Nm/A
$I_{dem}$	Demagnetization current	21.5 A
$f_{s}$	Execution frequency ^1^	8 kHz

^1^ The switching frequency, algorithm execution frequency, and sampling frequency are all 8 kHz.

**Table 2:** Compressor pressure.

Speed (rpm)	Suction Side (MPa)	Exhaust Side (MPa)
1200	0.5	2.1
1800	0.35	2.2
2400	0.33	2.5
3600	0.28	2.9

**Table 3:** FFT results at different speeds.

Speed (rpm)	Without	QPRC	ABRC	APSFSM
1200	38.79%	0.52%	5.66%	0.01%
1800	23.08%	0.17%	3.11%	0.05%
2400	10.78%	0.16%	1.41%	0.08%
3600	7%	0.22%	0.72%	0.08%

## Data Availability

The data are available upon reasonable request.
